# Biochemical Marker Assessment of Chronic Carbamazepine Exposure at Environmentally Relevant Concentrations in Juvenile Common Carp (*Cyprinus carpio*)

**DOI:** 10.3390/antiox11061136

**Published:** 2022-06-09

**Authors:** Xinyue Liang, Zsolt Csenki, Bence Ivánovics, Illés Bock, Balázs Csorbai, József Molnár, Erna Vásárhelyi, Jeffrey Griffitts, Árpád Ferincz, Béla Urbányi, András Ács

**Affiliations:** 1 Department of Freshwater Fish Ecology, Hungarian University of Agriculture and Life Sciences, Páter Károly u. 1., H-2100 Gödöllő, Hungary; liang.xinyue@uni-mate.hu (X.L.); ferincz.arpad@uni-mate.hu (Á.F.); 2 Department of Environmental Toxicology, Institute of Aquaculture and Environmental Safety, Hungarian University of Agriculture and Life Sciences, Páter Károly u. 1., H-2100 Gödöllő, Hungary; csenki-bakos.zsolt.imre@uni-mate.hu (Z.C.); ivanovics.bence@uni-mate.hu (B.I.); bock.illes@uni-mate.hu (I.B.); vasarhelyi.erna@uni-mate.hu (E.V.); jeffrey.griffitts@uni-mate.hu (J.G.); 3 Department of Aquaculture, Hungarian University of Agriculture and Life Sciences, Páter Károly u. 1., H-2100 Gödöllő, Hungary; csorbai.balazs@uni-mate.hu (B.C.); molnar.jozsef@uni-mate.hu (J.M.); urbanyi.bela@uni-mate.hu (B.U.)

**Keywords:** carbamazepine, oxidative stress, fish biomarker, common carp (*Cyprinus carpio*), chronic effects

## Abstract

Worldwide, the anticonvulsant drug carbamazepine (CBZ) is the most frequently identified pharmaceutical residue detected in rivers. Reported chronic effects of CBZ in non-target freshwater organisms, particularly fish, include oxidative stress and damage to liver tissues. Studies on CBZ effects in fish are mostly limited to zebrafish and rainbow trout studies. Furthermore, there are only a few chronic CBZ studies using near environmental concentrations. In this study, we provide data on subacute effects of CBZ exposure (28 days) to common carp (*Cyprinus carpio*), employing a set of biochemical markers of damage and exposure. CBZ was found to induce a significant change in the hepatic antioxidant status of fish subjected to 5 µg/L. Moreover, with increasing concentrations, enzymatic and non-enzymatic biomarkers of oxidative defence (catalase (CAT), superoxide dismutase (SOD), glutathione reductase (GR), DNA strand breaks)), toxicant biotransformation (ethoxyresorufin-o-demethylase (EROD), glutathione-S-transferase (GST)), and organ and tissue damage (lactate dehydrogenase (LDH), cetylcholinesterase (AChE)) were altered. The AChE, LDH, and lipid peroxidation (LPO) results indicate the occurrence of apoptotic process activation and tissue damage after 28 days of exposure to CBZ. These findings suggest significant adverse effects of CBZ exposure to common carp at concentrations often found in surface waters.

## 1. Introduction

Pharmaceuticals have received a lot of attention recently, being produced, consumed, and released into the environment in high amounts [1]. Removal or biodegradation of these compounds and their metabolites is very limited in wastewater treatment plants (WWTP) [2]. When released into the environment, pharmaceutical residues may bioaccumulate [3] and/or exert noxious effects on living organisms [4], particularly fish [5,6,7]. Being biologically active compounds that are intended to interact with specific processes and biochemical pathways in humans and animals, alterations in similar metabolic pathways in non-target organisms exposed to pharmaceuticals cannot be ignored [3].

Carbamazepine (CBZ) is an anticonvulsant drug prescribed worldwide for the treatment of bipolar disorder, trigeminal neuralgia, and psychomotor epilepsy [8]. Among psychiatric drugs, CBZ is one of the most commonly found pharmaceuticals in municipal WWTP effluents and urban impacted surface waters [9,10,11]. Anthropogenic activities serve as a continuous source of CBZ release to the environment [9]. CBZ is absorbed almost completely within the human gastrointestinal tract, and the majority (72%) of the received dose enters sewage systems as a component of human urine [12]. WWTPs are only able to remove 10% of the CBZ that enters the sewage system [13]. The remaining CBZ is released with the WWTP effluent into the environment where it undergoes a relatively slow degradation process with a permanency time of around 82 days in surface waters [14]. Accordingly, CBZ has been detected in surface waters worldwide in concentrations of 150 µg/L in South Korea [15], 12 µg/L in Europe [16], and 0.8 µg/L within the Danube River in Hungary [17,18].

In humans, CBZ has been shown to interact with potassium and sodium channels and, in addition, some signalling pathways [19]. It was also shown that CBZ modulates voltage-gated sodium channels, resulting in decreased neuronal activity [20]. Regarding the biota of freshwater habitats, some studies have been performed in the last decade to understand the lethal and sublethal effects of CBZ. Assessments included organisms such as algae, cladocerans, and fish [7,21,22,23].

Reported chronic effects include a decline in fecundity, decreased embryo production, and irregular oocytes. These chronic effects are likely due to altered sex steroid hormones in the case of zebrafish (*Danio rerio*) [24], suggesting CBZ can have endocrine-disrupting effects. Additional CBZ research in zebrafish has shown an increased time for first feeding action and total food ingestion, modulation of acetylcholinesterase (AChE) and liver glutathione-S-transferase (GST) activity, decreased catalase (CAT) and lactate dehydrogenase (LDH) activity, and DNA damage after 63 days of continuous CBZ exposure [7].

In common carp (*Cyprinus carpio*), Li et al. [23] reported reduced activity in superoxide dismutase (SOD), glutathione peroxidase (GPx), and glutathione reductase (GR) combined with increased lipid peroxidation (LPO) in the sperm of common carp following high CBZ concentration exposure (between 0.2 and 2 mg L^−1^) [23]. The same research showed increased lipid peroxidation in brain tissues of rainbow trout (*Oncorhynchus mykiss*) accompanied by decreased SOD and GR, while GPx and CAT presented a non-linear response over time with an increase then subsequent decrease in their activities [25]. Li et al. [23] showed that high concentrations of CBZ, between 0.2 and 2 mg/L, cause an increase in the degree of LPO and carbonylated proteins in addition to a reduction in the activity of SOD, GR, and GPx activity in *Cyprinus carpio* sperm after two hours of in vitro exposure. In a recent study, Gasca-Pérez et al. [26] reported increases in LPO, hydroperoxide, and protein carbonyl content with a decrease in the activity of antioxidant enzymes (SOD, GPx, CAT) after sub-acute (7 days) treatment with 2 mg/L of CBZ.

*Cyprinus carpio* is a convenient bioindicator, having appropriate sensitivity to xenobiotic exposure, adaptability to laboratory conditions, wide global distribution, and economic significance [26,27,28,29]. Most recent studies on CBZ effects in fish are mostly limited to zebrafish and rainbow trout [30]. Additionally, there is a lack of chronic CBZ studies using near environmental concentrations. Thus, our study provides data on the potential risk of CBZ to common carp through chronic exposure (28 days) at environmentally relevant concentrations using a set of biochemical markers of damage (DNAsb, LDH, LPO, VTG), and exposure (CAT, EROD, GST, GR, SOD).

## 2. Materials and Methods

### 2.1. Chemicals

CBZ (CAS 298-46-4) was purchased from Sigma-Aldrich (Darmstadt, Germany). All other reagents used in the study were of analytical grade.

### 2.2. Fish Maintenance

Common carp juveniles were kept in an individually designed recirculating fish housing system of the Department of Environmental Toxicology at the Hungarian University of Agriculture and Life Sciences (Gödöllő, Hungary). The fish were kept in 10 m^3^ tanks with constantly maintained water quality parameters (22 ± 2 °C, pH 7.8 ± 0.2, redox potential, 230 ± 2 mV, dissolved O2-level, 6.8 ± 1 mg/L) and a light:dark period of 14 h:10 h. The carp were fed 10 g/kg body weight AquaGarant Aquastart (Aqua Garant, Pöchlarn, Austria) pelleted feed (1.2–1.5 mm) two times a day.

### 2.3. Experimental Design

For the subacute, 28 days, juvenile fish test, males and females (weight 7.37 ± 1.35 g) were randomly distributed into fifteen experimental tanks, each containing 50 L of the test solution (nominal concentrations are as follows: 0, 1, 5, 50, or 100 μg/L of CBZ). Fifteen fish (3 replicates of 5 fish each) were used per treatment. The lowest and highest CBZ concentrations tested, 1 μg/L and 100 μg/L, were selected based on the recent paper by da Silva Santos et al. [7]. The carp were exposed for 28 days to the test solutions while also being fed a 10 g/kg body weight AquaGarant Aquastart (Aqua Garant, Pöchlarn, Austria) pelleted feed (1.2–1.5 mm) two times a day. The test media was completely renewed every three days. Water quality parameters were kept within the ranges described in the preceding “Fish maintenance” section. To ensure agreement between nominal and actual compound concentrations in the aquaria, water samples were analysed during the experimental period by LC–MS/MS. Water samples were collected from the test aquaria after 1 h and 36 h of renewing the test solutions. The mean concentration of CBZ in the water samples was consistently within 20% of the intended concentration.

On the 7th, 14th, and 28th day of exposure, five fish from each exposure concentration and replicate were sacrificed. The brain, liver, and intestine of each fish were isolated and stored in microtubes at −80 °C for later biochemical analyses.

### 2.4. Biochemical Determinations

Homogenization was performed using a small bead mill (TissueLyser LT, Qiagen, Germantown, MD, USA). Enzymatic activities were evaluated in triplicate using a Thermo Varioskan™ LUX multimode microplate reader at 25 °C (Thermo Fisher Scientific, Waltham, MA, USA).

Fish intestines and approximately half of the liver tissue were homogenised in a general buffer (25 mM Hepes-NaOH, 130 mM NaCl, 1 mM EDTA, 1 mM dithiothreitol, pH = 7.4) at a weight to volume ratio of 1:5. Subsamples of homogenates were frozen at −80 °C for lipid peroxidation analysis (LPO), DNA strand breaks (DNAsb), vitellogenin-like proteins (Vtg), and ethoxyresorufin-o-demethylase (EROD). The remaining liver tissues were homogenised in 100 mM of phosphate buffer (pH = 7.4, KCl 100 mM, EDTA 1 mM, dithiothreitol (DTT), 0.5 M sucrose, and 40 µg/mL aprotinin) and centrifuged at 12,000× *g* for 30 min at 4 °C. The supernatants (S12) were collected, and aliquots were kept at −80 °C until GR, GPx, GST, CAT, LDH, and SOD analyses could be conducted. Brain tissues were homogenised in 0.1 M phosphate buffer (pH = 7.2; 1:10 *w*/*v*) and subsequently centrifugated at 6000× *g* for 3 min at 4 °C. The protein concentration of the samples was determined, in triplicate, by the Bradford method [31], adapted to microplate, using bovine serum albumin as a standard. The absorbance was recorded at 595 nm after an incubation period of 15 min.

Lipid peroxidation was evaluated based on the formation of malonaldehyde in tissue homogenates by the thiobarbituric acid method elaborated by Wills [32]. A 150 μL homogenate was mixed with 300 μL of 10% trichloroacetic acid containing 1 mM FeSO_4_ and 150 μL of 0.67% thiobarbituric acid. The mixture was heated to 80 °C for 10 min, then precipitates were removed by centrifugation (10,000× *g* for 10 s). The supernatant was subjected to fluorescence measurement at 516 nm excitation/600 nm emission. Blanks and standards of tetramethoxypropane were prepared in a homogenization buffer. Results were expressed as μmoles of thiobarbituric acid reactants per milligramme of homogenate protein.

The determination of AChE activity was carried out according to the method of Ellman et al. [33] and adapted to microplate [34]. A 96-well microplate was loaded with 3 replicates of 50 μL of homogenate supernatant and 250 μL of a solution made with 0.075 M acetylthiocholine iodide and 10 mM 5,5 dithio-bis(2-nitrobenzoic acid) in phosphate buffer (0.1 M, pH = 7.2). In assay blanks, samples were substituted with phosphate buffer and electric eel acetylcholine esterase was used as a positive control. Absorbance was measured every minute at 414 nm for a total of 15 min. Enzymatic activity was calculated from the slope of the absorbance curve and was expressed in units (U) per mg of protein content (1 U being 1 μmol of substrate hydrolysed/min).

CAT activity was measured in triplicate following the method of Aebi [35]. Decreases in the absorbance of a 50 mM H_2_O_2_ solution (ε = −0.0436 mM^−1^ cm^−1^) in 50 mM phosphate buffer (pH 7.8) and 10 µL of tissue supernatant (S12) were continuously recorded at 240 nm at 10 s intervals for 1 min. The results were expressed as U/mg protein; a unit of CAT was defined as the amount of enzyme that catalysed the dismutation of 1 mmol of H_2_O_2_/min.

GST activity was determined by the method of Habig et al. [36], adapted to microplate, whereby a solution of 100 mM glutathione (GSH) in phosphate buffer (pH = 6.5) and a second solution of 60 mM 1-chloro-2,4-dinitrobenzene (CDNB, ε = 9.6 mM^−1^ cm^−1^) in ethanol was prepared just before the assay. The reaction mixture consisted of phosphate buffer, GSH solution, and CDNB solution in a proportion of 4.95 mL (phosphate buffer): 0.9 mL (GSH): 0.15 mL (CDNB). In the microplate, 0.2 mL of the reaction mixture was added to 0.1 mL of the sample (S12), and the GST activity was measured immediately at 20 s intervals, at 340 nm, for a period of 5 min. GST from equine liver was used as a positive control. Enzymatic activity was calculated from the slope of the absorbance curve and was expressed in units (U) per mg of protein content (1 U being 1 μmol of substrate hydrolysed/min).

GR activity was measured by the decrease of NADPH at 340 nm for 1 min and expressed as µM/mg prot./min according to Carlberg and Mannervik [37]. The reaction medium contained 100 mM phosphate buffer (pH = 7.4), 0.1 mM NADPH and 30 µL of the supernatant (S12). GR activity was expressed as U per mg of protein (a U corresponding to 1 µM NADPH hydrolysed/min).

LDH activity was measured according to the methodology described by Vassault [38], adapted to microplate by Diamantino et al. [39]. A volume of 25 μL samples and 125 μL of a 300 μM reduced nicotinamide adenine dinucleotide solution (NADH) were added to 20 μL of a 4.5 mM pyruvate solution. Reading of the microplates was performed at 340 nm for intervals of 40 s over a period of 5 min, following a decrease in absorbance resulting from the oxidation of NADH. LDH activity was expressed as U per mg of protein (a U corresponding to 1 µM NADPH hydrolysed/min).

EROD activity was determined according to the method described in Burke and Mayer [40]. Subsamples of tissue homogenates were centrifuged at 12,000× *g* for 30 min at 4 °C. Fifty microliters of the resulting supernatant were incubated at 30 °C for 60 min in a 150 µL mixture containing 100 mM phosphate buffer (pH = 7.4), 100 μM reduced NADPH, and 10 μM 7-ethoxyresorufin. The reaction was initiated by the addition of NADPH and halted by the addition of 100 μL of 0.5 M NaOH. The resultant 7-hydroxyresorufin was determined by fluorometry at 520 nm excitation/590 nm emission wavelengths. Calibration was performed with serial dilutions of 7-hydroxyresorufin. Results were expressed as pM 7-hydroxyresorufin generated in one minute per mg of protein.

DNA strand breaks were quantified by an adaptation of the alkaline precipitation assay of Olive [41]. A 25 μL tissue homogenate was mixed with 200 μL of 2% SDS containing 10 mM EDTA, 10 mM Tris-base, and 40 mM NaOH. The resulting mixture was then shaken for 1 min. Two hundred microliters of 0.12 M KCl were added to the mixture and then heated at 60 °C for 10 min, mixed by inversion, and then cooled at 4 °C for 30 min. Finally, the total mixture was centrifuged at 8000× *g* for 5 min at 4 °C. Fifty microliters of the resulting supernatant were then added to 150 μL of Hoechst dye (1 μg mL^−1^, in buffer containing 0.4 M NaCl, 4 mM sodium cholate and 0.1 M Tris-acetate, pH 8.5–9, and mixed for 5 min on a plane shaker). Fluorescence was measured at 360 nm excitation/450 nm emission wavelengths. Sample blanks contained identical constituents, with 25 μL Hepes buffer replacing the tissue homogenate. A salmon sperm DNA standard (Sigma) was used for DNA calibration and the results were expressed as μg DNA_sb mg^−1^ protein.

Vitellogenin-like proteins (Vtg) were determined in the 12,000 g microsomal fraction following the alkali-labile phosphate (ALP) method developed by Blaise et al. [42]. Two hundred microliters of sample homogenate were mixed with 54 μL of acetone (35% final concentration) for 10 min and centrifuged at 10,000× *g* for 5 min. The retained pellet was then dissolved in 50 μL of 1 M NaOH and mixed for 30 min at 60 °C. The total phosphate was then determined by the colorimetric phosphomolybdenum method developed by Stanton [43]. To a 20 μL sample, 125 μL H_2_O, 5 μL 100% TCA, 25 μL of molybdate reactive, and 25 μL of 1% ascorbate were added and then mixed for 10 min. The absorbance was read at 815 nm and 444 nm. Rainbow trout vtg was used for calibration and aliquots of NaOH (1 M) were used as blanks. Vtg levels were expressed as μmoles of ALP per milligramme of protein.

Total SOD activity was measured using the xanthine oxidase/cytochrome c method proposed by Crapo et al. [44] in the S12 fraction. Cytochrome c reduction by superoxide anions, generated by the xanthine oxidase/hypoxanthine reaction, was detected at 550 nm at ambient temperature. Enzyme activity was expressed as U/mg protein; a unit of SOD was defined as the amount of sample producing 50% inhibition under the assay conditions. The reaction mixture contained 46.5 mM KH_2_PO_4_/K_2_HPO_4_ (pH = 8.6), 0.1mM EDTA, 195 mM hypoxanthine, 16 mM cytochrome c, and 2.5 mU xanthine oxidase. The enzymatic activity was calculated from the slope of the absorbance curve.

The values of each biomarker were normalized against the protein content of either the whole homogenate or supernatant [31].

### 2.5. Statistical Analysis

All data were analysed using the statistical software package OriginPro, version 2019 (OriginLab Corporation, Northampton, MA, USA). To examine the interactive effects of different CBZ concentrations and exposure times on biochemical markers, a two-way analysis of variance (ANOVA) was used, where time (t = 7; t = 14; t = 28 days), treatment (control, 1, 5, 50, 100 µg/L), and their interaction were categorical predictor factors, while the measured biomarkers were considered as dependent variables. When the interaction of CBZ concentration and the exposure time was detected, a one-way ANOVA was conducted to examine the effects of one main factor at a specific level of the other main factor. Factors determined to be significant were further analysed using a post-hoc Tukey test for multiple comparisons at a significance level of 0.05 (*p* < 0.05). Before statistical analyses, raw data were diagnosed for normality of distribution and homogeneity of variance with the Kolmogorov-Smirnov test and Levene’s test, respectively.

## 3. Results

None of the fish, control, and CBZ exposed, died during the experimental assay at any of the tested conditions.

### 3.1. AChE Activity

**Figure 1:**
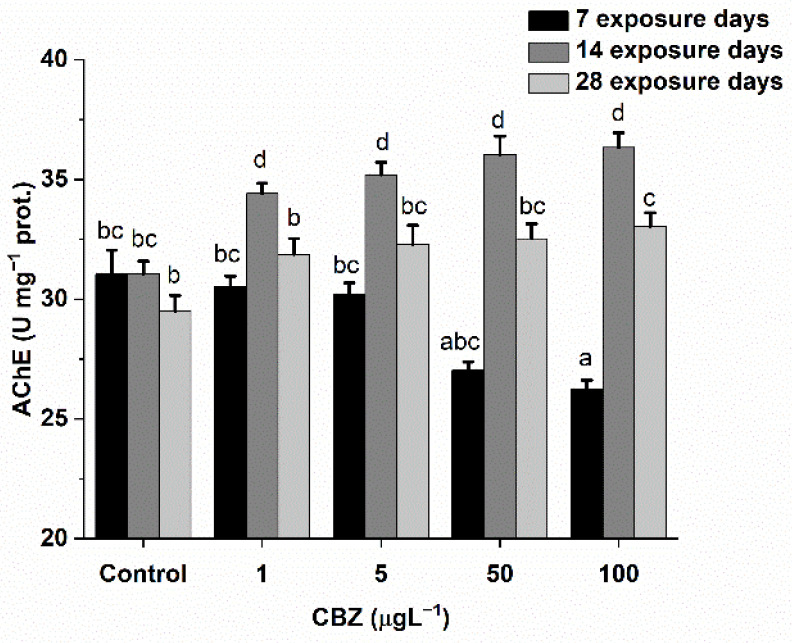
Changes in AChE activity in the brain of *Cyprinus carpio* exposed to CBZ for 7, 14, and 28 d. Data are expressed as mean ± standard deviation of three replicates (*n* = 3). Different letters designate significant differences at *p* < 0.05 after a two-way ANOVA followed by Tukey’s post-hoc test.

The results of AChE activity in *Cyprinus carpio* after exposure to CBZ are shown in Figure 1. Compared to the controls, exposure to CBZ induced time-dependent changes in AChE activities. After seven days, AChE activity significantly (*p* < 0.05) decreased at concentrations above 5 µg/L (50, 100 µg/L). Conversely, AChE activity increased as the CBZ concentration increased after 14- and 28-day exposures. This increasing trend in AChE activity after the first week was statistically (*p* < 0.05) confirmed to be time-dependent and not concentration-dependent.

### 3.2. Biotransformation Enzymes

**Figure 2:**
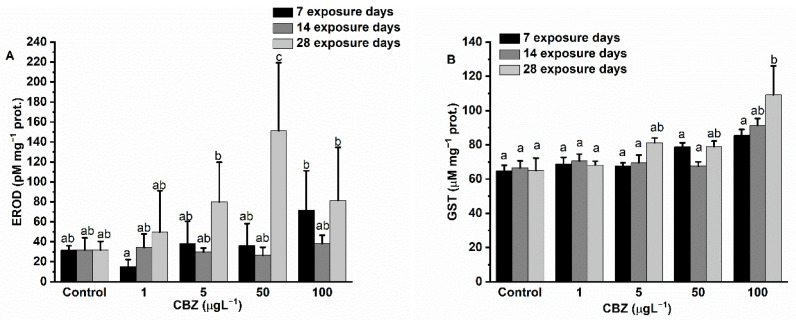
Effect of CBZ on level of hepatic EROD (**A**), and hepatic GST (**B**) in liver of common carp. Data are means ± S.D., *n* = 3. Columns sharing the same superscript letter indicate no significant differences after a two-way ANOVA followed by Tukey’s post-hoc test (*p* > 0.05).

After 7 days of exposure, hepatic EROD activity levels in the test animals showed a concentration-dependent increase (peaking at a concentration of 100 µg/L CBZ) after first starting with a non-significant decrease in the case of 1 µg/L CBZ exposure as compared to controls. Fourteen days of CBZ exposure resulted in no significant differences being detected as compared to the controls and also between different CBZ concentrations. By the 28th day of CBZ exposure, a significant (*p* < 0.05) increase in EROD activity was measured in exposure concentrations above 1 µg/L (5, 50, and 100 µg/L) CBZ (Figure 2A). Interestingly, EROD activity levels measured after 28 days of exposure to 100 µg/L CBZ appeared lower than in the case of 50 µg/L measured activities; however, this difference was not significant (*p* < 0.05).

While GST activity values showed an increasing, concentration-dependent tendency during the 28-day exposure; however, a significant elevation in GST activity was measured only at the highest exposure concentration (100 µg/L) after the fourth week.

### 3.3. Antioxidant Defence

**Figure 3:**
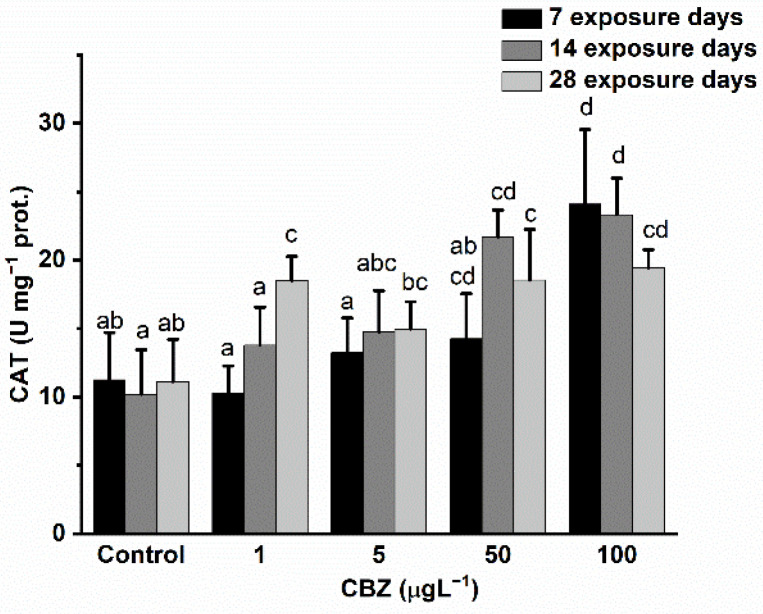
Changes in the CAT activity in the liver of *Cyprinus carpio* exposed to CBZ for 7, 14, and 28 d. Data are expressed as mean ± standard deviation of three replicates (*n* = 3). Same letters designate no significant differences at *p* < 0.05 after a two-way ANOVA followed by Tukey’s post-hoc test.

CAT activity changes followed a time- and concentration-dependent pattern during the 28-day CBZ exposure. Significant (*p* < 0.05) differences, as compared to controls, first appeared in the case of 100 µg/L CBZ concentration after 7 days of exposure and again in the 50 µg/L and 100 µg/L CBZ concentrations after 14 days of exposure. After 28 days, a significant increase in CAT activity was measured at all CBZ exposure concentrations (1, 5, 50, and 100 µg/L) (Figure 3).

**Figure 4:**
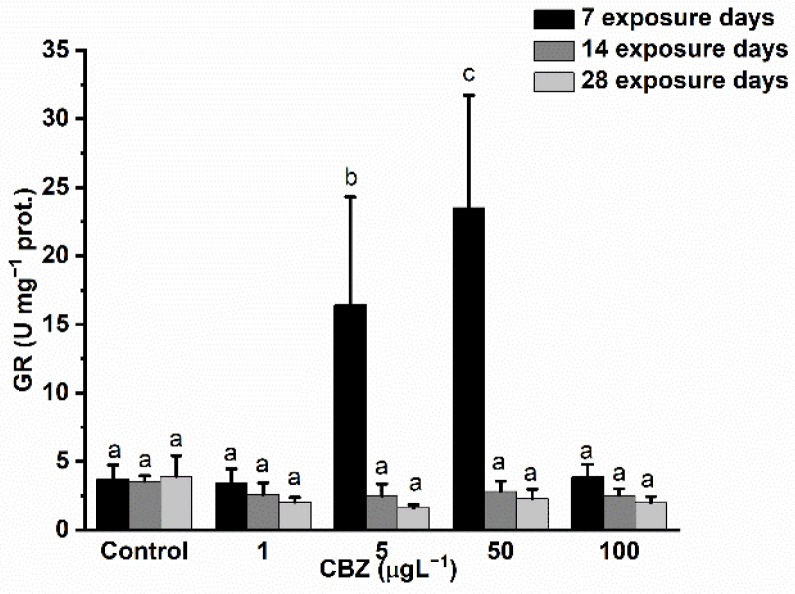
Changes in the GR activity in the liver of *Cyprinus carpio* subjected to CBZ for 7, 14, and 28 d. Data are expressed as mean ± standard deviation of three replicates (*n* = 3). Different letters designate significant differences at *p* < 0.05 after a two-way ANOVA followed by Tukey’s post-hoc test.

Measurements of GR activity showed a significant and concentration-dependent increase as compared to control values after 7 days of 5 and 50 µg/L CBZ exposure. In the case of 100 µg/L CBZ concentration, GR activity dropped back to similar values to the controls. After 14 days of CBZ exposure, GR activity levels remained slightly lower as compared to controls, showing time and concentration dependency, but the decrease in GR activity proved to not be significantly different to the controls (Figure 4).

**Figure 5:**
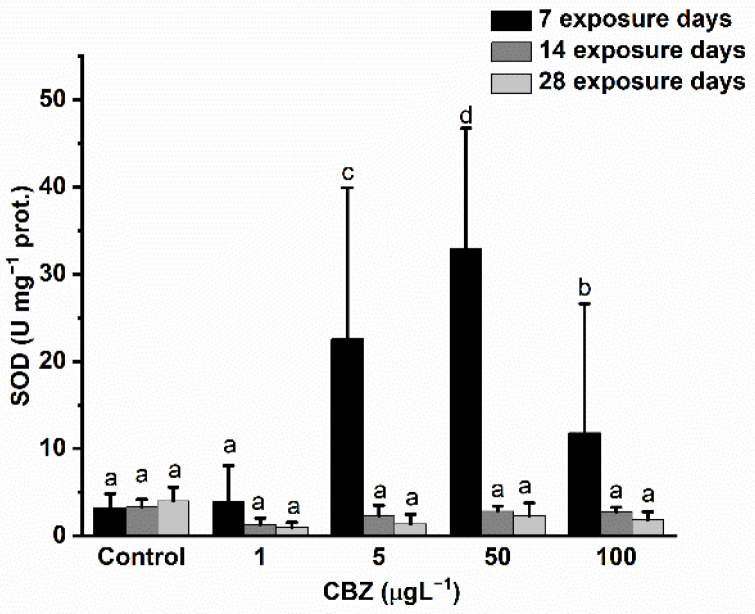
Changes in the SOD activity in the liver of *Cyprinus carpio* exposed to CBZ for 7, 14, and 28 d. Data are expressed as mean ± standard deviation of three replicates (*n* = 3). Different letters designate significant differences at *p* < 0.05 after a two-way ANOVA followed by Tukey’s post-hoc test.

SOD activity changes exhibited a very similar response pattern to GR activity. After seven days of 5, 50, and 100 µg/L CBZ exposure, a significant (*p* < 0.05) and concentration-dependent increase was detected. After 100 µg/L CBZ concentration exposure, activity was significantly lower compared to 5 and 50 µg/L CBZ concentration exposure but higher than control values. After 14- and 28-days exposure in all groups treated with CBZ, a decrease in SOD activity was detected as compared to control values, but this decrease was not significant (*p* < 0.05) (Figure 5).

**Figure 6:**
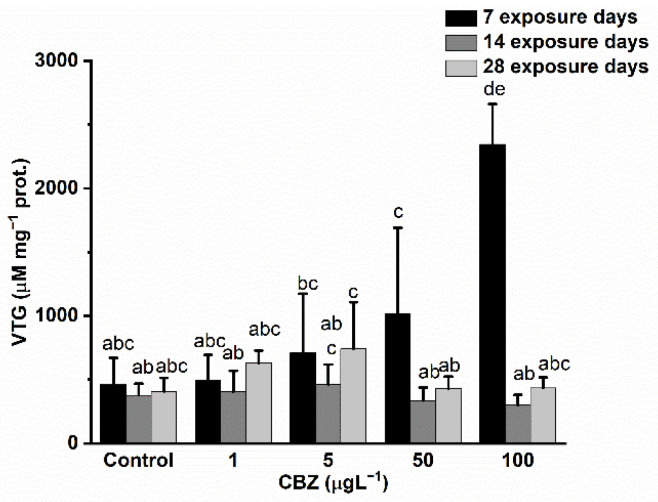
Changes in the VTG levels in the gonads of *Cyprinus carpio* exposed to CBZ for 7, 14, and 28 d. Data are expressed as mean ± standard deviation of three replicates (*n* = 3). Different letters designate significant differences at *p* < 0.05 after a two-way ANOVA followed by Tukey’s post-hoc test.

VTG levels increased following a concentration-dependent pattern after the first seven days of exposure. A significant (*p* < 0.05) elevation of VTG content in the samples was detected in fish subjected to 50 and 100 µg/L CBZ exposure. After 14 days, VTG levels returned to a level similar to the control groups, but no significant differences were observable. After the 28th exposure day, no significant (*p* < 0.05) elevation in VTG levels was detected compared to the control groups (Figure 6).

### 3.4. Damage Markers

**Figure 7:**
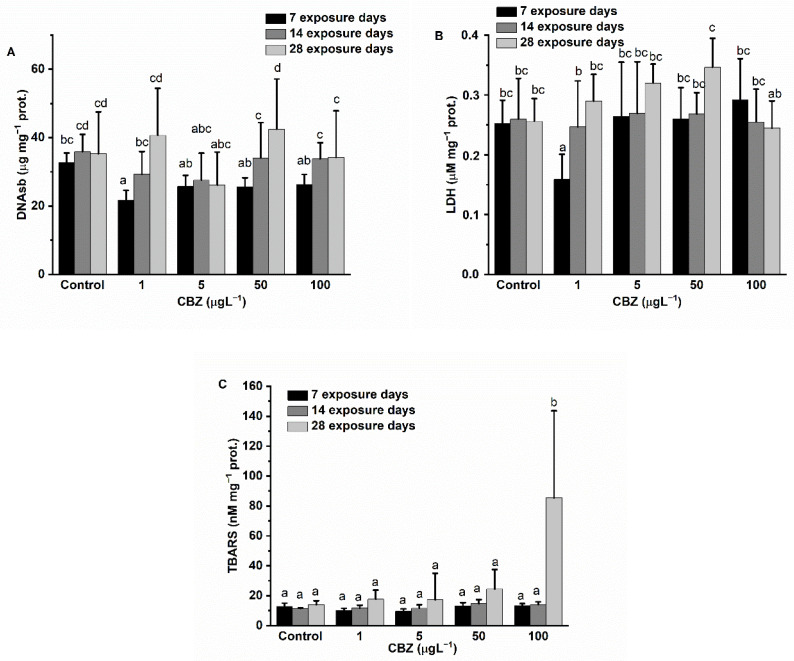
Changes in the DNA strand breaks (**A**), LDH activity (**B**), and level of lipid peroxidation (**C**) in the liver of *Cyprinus carpio* exposed to CBZ for 7, 14, and 28 d. Data are expressed as mean ± standard deviation of three replicates (*n* = 3). Same letters designate no significant differences at *p* < 0.05 after a two-way ANOVA followed by Tukey’s post-hoc test.

The DNAsb measured in liver samples was slightly decreased as compared to controls during the first two weeks of exposure to CBZ, with differences in measured levels diminishing by the fourth week of exposure. Differences (decrease) in experimental DNAsb levels compared to the control groups proved significant only with 1 µg/L CBZ concentration exposure after 7 days of exposure. Statistically, there was no time- or concentration dependence (Figure 7A).

LDH activity in the experimental groups showed a significant decrease compared to the measured activity in the control groups at 1 µg/L concentration after 7 days of exposure. However, at higher exposure concentrations (5, 50, and 100 µg/L) of CBZ, there were no significant changes measured. After 14 days of exposure, no activity changes, compared to controls, were detectable. After 28 days of exposure, an increasing trend in LDH activity was observed. In this group, LDH activity peaked at 50 µg/L CBZ concentration, but at 100 µg/L CBZ concentration, LDH levels showed no significant difference compared to control levels (Figure 7B).

LPO levels showed no difference compared to control measured values until after 28 days of exposure. On the 28th exposure day, LPO levels markedly increased, significantly peaking at 100 µg/L CBZ (*p* < 0.05) (Figure 7C). Although a concentration-dependent pattern was visible in the 28-day exposure group, statistically significant differences as compared to controls were only confirmed in the highest exposure concentration (100 µg/L CBZ).

## 4. Discussion

In a recent study, CBZ was determined to be the most frequently identified and the 9th highest in concentration among pharmaceutical residues detected in rivers worldwide [11]. CBZ has been shown to exert harmful chronic effects on non-target organisms, particularly fish, even in very low concentrations [7,45]. In our study, the sub-chronic effects of CBZ were assessed in *Cyprinus carpio* at environmentally relevant concentrations.

The AChE inhibition observed after 7 days of CBZ exposure in this study is in agreement with the significant reduction in AChE activity previously found in *R. philippinarum* [46], in the monogonont rotifer (*Brachionus koreanus*) [47], and in crucian carp (*Carassius carassius*) [45] after short-term (<7 days) exposure to CBZ. Reduced AChE activity is attributed to neurotoxic agents, and it is a commonly applied biochemical marker of neurotoxic environmental pollutants [48]. However, in this study, following 14- and 28-day exposure to CBZ, a time and concentration-dependent increase was observed in AChE activity. Increased AChE activity is often associated with the production of free radicals and oxidative stress [49] and ongoing apoptotic processes in the test organisms [50,51]. Yan et al. [52] showed that CBZ, at environmentally relevant concentrations (1, 10, 100 µg/L), causes apoptosis in the liver of Chinese rare minnows (*Gobiocypris rarus*). Accordingly, the elevated AChE activity measured in this study may be a consequence of apoptotic processes. Elevated AChE activity causes a fast degradation of the neurotransmitter acetylcholine and a subsequently decreased stimulation of acetylcholine receptors affecting the cognitive functions of the organisms [53]. Additionally, a similar increase in AChE activity was found after 63 days of CBZ exposure in zebrafish [7].

The phase I biotransformation enzyme EROD is a member of the aryl hydrocarbon (AhR) receptor-regulated P450-dependent mono-oxygenase CYP1A family, and is widely used as a biomarker in fish for screening the uptake of environmental organic pollutants [54]. The significant increase in EROD activity after 28 days of exposure to 50 and 100 µg/L CBZ concentrations indicates that CYP1A enzymes were biosynthesized to detoxify and metabolise CBZ. These results are in accordance with a previous study with *Carassius carassius*, where 2 and 10 µg/L CBZ concentrations were shown to induce hepatic EROD activity after 1, 4, and 7 days [45]. Our observation of a decrease in EROD activity after 28 days of exposure to a concentration of 100 µg/L CBZ, as compared to fish exposed for 28 days to 50 µg/L CBZ, may be attributed to liver damage, as proposed in the case of AChE.

GST, a member of the Phase II biotransformation enzyme group, is implicated in the conjugation of xenobiotics with glutathione, increasing their solubility and excretion [4]. The significant increase in GST activity measured during our study suggests an oxidative stress-induced adaptive response or, alternatively, the conjugation and excretion processes of CBZ in the liver of common carp. This result is in agreement with a previous study performed by Nkoom et al. [45], where 2 and 10 µg/L CBZ concentration exposure was shown to cause a significant increase in the activity of GST in the liver of *Carassius carassius* after 1, 4, and 7 days. The data obtained for the biotransformation enzymes (EROD and GST activities) in our study indicates that CBZ was both biotransformed and metabolised in the liver of common carp.

Reactive oxygen species (ROS) are present at low concentrations in organisms with a normal functioning metabolism. High concentrations (a possible result of the presence of some xenobiotics) may inflict adverse effects on cellular components, such as lipids, proteins, carbohydrates, and DNA. Therefore, the equilibrium of the redox state is fundamental for the proper functioning of organisms [55]. This vital balance is maintained by antioxidants (e.g., reduced glutathione), antioxidant enzymes (e.g., SOD, CAT, GPx, and GR), and enzymes able to reduce the oxidised form of glutathione (e.g., GR) [55]. In this study, the alterations in antioxidant defence system enzyme activities measured in the liver of common carp illustrate the strong oxidative stress effect of CBZ. After an initial increase in the activity of SOD and GR during the first seven days of CBZ exposure, their activity dropped below levels measured in the control group after 14 days and remained suppressed in the 28-day treatment group. CAT activity increased significantly through the 28 days of exposure compared to the activity levels measured in the control group. These results are in accordance with preceding studies. For instance, several previous studies reported significantly increased SOD, CAT, and GR activities after short-term (<7 days) exposure to CBZ in rainbow trout [25] and *Carassius carassius* [45]. The initial increase in the activity of antioxidant enzymes could be explained by elevated ROS concentrations actuating the antioxidant system and SOD activity in the tissues of fish in order to initiate the dismutation of ROS derived from drugs (such as superoxide anion radical O_2_^−•^) to molecules which are less toxic (such as H_2_O_2_). In prior studies, increasing levels of products from this process, together with H_2_O_2_ produced by oxidase enzymes (e.g., xanthine oxidase, amino acid oxidase, and NAD(P)H oxidase), upregulated CAT activity and excess H_2_O_2_ was converted to H_2_O and O_2_ [55,56,57]. The cooccurring increase in the activity of GR subserves the conversion of oxidised glutathione (GSSG) to reduced GSH; reduced GSH can directly scavenge ROS and is subsequently reduced to GSSG in an energy-demanding process utilising NADPH [57]. The detected drop after an initial increase in SOD and GR enzymes may be attributed to the following: lipid peroxidation and the direct attack of reactive oxygen species, proteins decreasing ROS [58], or an energy (NADPH) shortage following prolonged exposure to CBZ [57,58]. CAT activity may have remained at a higher level due to H_2_O_2_ originating from sources other than SOD activity.

Low-intensity oxidate stress can induce cells to produce antioxidant enzymes that are able to eliminate ROS, while severe oxidate stress can overwhelm these protective enzymes, resulting in oxidative damage of cell components like lipids, proteins, and even DNA [55]. TBARS is the most widely used indicator of lipid peroxidation (LPO) triggered by oxidative stress in fish [59]. Our results showed a significant elevation of TBARS only at the end of the tests in the case of the highest applied CBZ concentration (100 µg/L); however, the continuous elevation of TBARS levels in our data as compared to control values hints at an ever-growing oxidative stress pressure due to a malfunction of the antioxidant defence system. This finding corresponds to other studies, for example, Li et al. [58], that reported oxidative stress and elevated TBARS levels in rainbow trout after 21- and 48-day exposure to 20 and 200 µg/L CBZ.

CBZ was shown to cause alterations in the genetic material of Chinese rare minnows (*Gobiocypris rarus*) after 28 days of 1, 10, and 100 µg/L CBZ exposure [52]. In the present study, strand break levels showed a slight decrease during the 28-day tests in common carp compared to control groups. The observed lower level of DNA strand breaks could be attributed to repair or recovery mechanisms [60] initiated by oxidative stress effects. An inhibitory effect on cell division may also be involved in the observed responses [61].

The LDH is a widely used marker of organ and tissue damage reflecting metabolic activity (e.g., carbohydrate metabolism), as well as structural and morphological alterations of tissues that are closely associated with pathological processes [62]. In our study, LDH activity was slightly increased in 50 µg/L CBZ concentration experimental groups after 28 days of exposure and decreased in 100 µg/L CBZ groups, although these changes were not significantly different from control activity values. Increased LDH activity in the liver and gills of common carp caused by 5700 µg/L CBZ exposure from 7 to 28 days was previously attributed to metabolic changes and tissue hypoxia due to the disruption of respiratory epithelium, resulting in a decrease in oxidative metabolism [63]. In another study, da Silva Santos et al. [7] reported elevated LDH levels in zebrafish after 63 days of 10,000 µg/L CBZ exposure and no change after 10 µg/L CBZ concentration. Our results agree with the above-mentioned studies and may be attributed to metabolic changes in liver cells. Our observed drop in LDH activity in the 100 µg/L, 28-day CBZ exposure group as compared to the LDH activity peak in the 50 µg/L CBZ group may also provide evidence for apoptotic damage in the liver tissues, as is also suggested in our results from the AChE, EROD, and antioxidant enzyme analyses.

CBZ was previously shown to have negative effects on fish reproduction. For instance, Li, et al. [23] showed that exposure to CBZ may inflict oxidative stress in common carp spermatozoa and impair sperm quality parameters. Da Silva Santos et al. [7] have shown that CBZ affects the reproductive success of zebrafish through the induction of pre-ovulatory follicles and increases the occurrence of atretic oocytes after 63 days of exposure to 10 and 10,000 µg/L CBZ concentrations. Moreover, in their study, histopathological analyses of ovarian tissues showed a significant increase in the proportion of vitellogenic follicles. The authors suggested that CBZ may have similar effects as other non-steroidal pharmaceuticals and pointed out that atretic oocytes are often reported as a toxic effect on reproduction caused by estrogenic compounds. Vitellogenin (VTG) from fish is a glycolipophosphoprotein produced in the liver and its production is induced by 17β-estradiol or compounds that are capable of interacting with the estrogen receptor [64]. In this study, significant increases in VTG levels were measured in fish subjected to 100 µg/L CBZ after seven days as compared to control values. In addition, our findings showed a statistically significant concentration dependence on VTG levels. This finding also corroborates the suggestion that CBZ’s toxic mechanistic routes may be similar to estrogenic compounds.

Preceding researches showed significant alteration of oxidative stress marker enzyme activities (SOD, CAT) and damage markers (LPO, DNA, LDH) after acute (<7 days) exposure to elevated concentrations (>200–10,000 µg/L) of CBZ in rainbow trout [25], *Carassius carassius* [45] and common carp spermatozoa [23]. Here, stress marker results after seven days of environmental relevant CBZ concentration exposure show a very similar pattern, as follows: antioxidant enzyme activities were elevated, and damage markers (DNAsb, LPO, and LDH) were not affected. After prolonged exposure to CBZ, our results hinted at the damage of cell components and apoptotic processes even in the case of the lowest exposure concentration (1 µg/L). Thus, the chronic adverse outcomes inflicted by exposure to environmentally relevant concentrations of CBZ observed in this study may be attributed mostly to prolonged oxidative stress effects.

## 5. Conclusions

In summary, chronic exposure to environmentally relevant concentrations of CBZ inflicted biochemical and, presumably, physiological effects in common carp. In this study, fish subjected to 5 µg/L of CBZ exhibited a significant change in hepatic antioxidant status. With increasing CBZ concentrations, enzymatic and non-enzymatic biomarkers of oxidative defence (CAT, SOD, GR, DNAsb), toxicant biotransformation (EROD, GST), and organ and tissue damage (LDH, AChE) were altered. The AChE, LDH, and LPO results are suggestive of apoptotic processes and tissue damage after 28 days of exposure to CBZ. The findings of the present study suggest significant adverse effects of CBZ on common carp at concentrations often found in surface waters.

## Data Availability

Data is contained within the article.
